# Reply to Fitzgerald et al.: Mischaracterizing our findings; the cognitive profile of elite footballers remains robust

**DOI:** 10.1073/pnas.2521662123

**Published:** 2026-02-18

**Authors:** Leonardo Bonetti, Torbjörn Vestberg, Reza Jafari, Debora Seghezzi, Martin Ingvar, Morten L. Kringelbach, Alberto Filgueiras, Predrag Petrovic

**Affiliations:** ^a^Department of Clinical Medicine, Center for Music in the Brain, Aarhus University and The Royal Academy of Music, Aarhus, Aalborg 8000, Denmark; ^b^Centre for Eudaimonia and Human Flourishing, Linacre College, University of Oxford, Oxford OX39BX, United Kingdom; ^c^Department of Psychiatry, University of Oxford, Oxford OX37JX, United Kingdom; ^d^Department of Psychology, University of Bologna, Bologna 40127, Italy; ^e^Department of Clinical Neuroscience, Karolinska Institutet, Stockholm 17177, Sweden; ^f^College of Psychology, School of Health and Applied Sciences, Central Queensland University, Cairns 40939, Australia; ^g^Department of Psychology, School of Education and Social Sciences, Rio de Janeiro State University, Riode, Janeiro 24435- 000, Brazil

Fitzgerald, Jakobsson, and Brodeur (FJB) (1) perform additional analyses addressing a narrower and different question compared to our original study (2) and use this procedure to criticize our claims. This is a rather concerning approach. More specifically, we investigated whether elite professional footballers differ from nonathlete individuals on a range of cognitive and personality measures, using both normative benchmarks and matched controls. In contrast, FJB compare national-team players (n = 23) with other first-division professionals (n = 28), a within-elite comparison we neither conducted nor proposed. Suggesting that small, nonsignificant differences between these subgroups undermine our findings is logically flawed. Detecting subtle differences within elite populations might require larger samples and is irrelevant to our primary question: whether elite footballers differ from the general population. Even if future work found no such intraelite differences, this would not challenge our core conclusion.

FJB speculate that Brazilian controls dropped out of school due to cognitive disadvantage, while players left to train professionally. There is no evidence for this claim. In fact, our controls performed on par with standardized D-KEFS norms: All subtest means approximated the normative score of 10, indicating cognitive representativeness. Recruiting higher-educated controls would have introduced more substantial confounds, as education level correlates with executive function (3–5).

Moreover, FJB refer to national statistics on average educational attainment in Brazil reported for 2024. However, the data used in our study were collected earlier. Importantly, the same reference cited by FJB (6) explicitly documents a steady year-by-year increase in educational attainment in Brazil. In addition, it is also reported that women, on average, attain higher levels of education than men. Consequently, comparisons based on aggregated 2024 data that combine males and females are not directly applicable to our sample, which consists exclusively of males and was recruited at an earlier time point. In the absence of sex-specific historical data corresponding to the period of data collection, extrapolating from recent mixed-sex statistics to infer that our control group represents individuals with unusually low educational attainment is not well justified.

We thank FJB for identifying a wording error: The tests in table 1 were one-sample *t* tests against a fixed mean, not two-sample tests. However, the analysis itself is valid and fully documented in our open source code. The reference mean of 10 is not arbitrary; it is the standard D-KEFS scaled-score mean, derived from a US-sample of approximately 1,750 individuals stratified on age, sex, ethnicity, and education and supported by subsequent validations (7–9). Such value is currently used worldwide since more recent analyses by the producer of the tests suggest that this norm has not changed substantially since the first study (10).

Finally, FJB argue that our machine learning model fails to distinguish between elite subgroups. But our model was designed to differentiate elite players from nonathletes, achieving 97% accuracy. It was never intended to detect subtle within-elite differences and repurposing it for such a task misrepresents our goals and methodology.

In sum, FJB’s critique addresses a narrower and different question using an underpowered design and use that to criticize our original findings, which remain robust and well-supported. We appreciate the opportunity to clarify these points.
